# Assessment of awareness on consumption of irradiated foods among Saudi population using a validated psychometric scale

**DOI:** 10.3389/fpubh.2024.1387219

**Published:** 2024-05-30

**Authors:** Nasser Shubayr

**Affiliations:** Department of Diagnostic Radiography Technology, College of Applied Medical Sciences, Jazan University, Jazan, Saudi Arabia

**Keywords:** irradiated foods, public awareness, Saudi Arabia, cross-sectional study, demographic factors

## Abstract

Despite the application of food irradiation for enhancing food safety, many consumers lack an understanding of its fundamental principles, often misinterpreting the information and exhibiting negative perceptions toward foods treated with ionizing radiation. This study focuses on evaluating public awareness regarding the consumption of irradiated food within Saudi Arabia, utilizing the Awareness Scale on Consumption of Irradiated Foods (ASCIF), a developed and validated tool. The ASCIF encompasses four constructs: concepts, awareness, labeling, and safety concerning irradiated foods. The average scores for each subscale and the aggregate ASCIF score were determined, with the analysis incorporating both descriptive and inferential statistical methods. The study's sample of 712 individuals predominantly consisted of females (53.37%), individuals aged 18–30 years (55.62%), those holding a bachelor's degree or higher (70.79%), participants earning less than SAR 5000 (42.70%), students (37.08%), and singles (66.85%). The overall mean scores for each category were as follows: safety (2.87 ± 0.92), concept (3.18 ± 0.79), label (3.44 ± 1.15), and awareness (2.68 ± 1.03). The overall mean score for the ASCIF was 3.02 ± 0.81, a diverse spectrum of awareness, with the majority of participants (62.92%) exhibiting intermediate awareness, while 17.98% displayed poor awareness, and 19.10% demonstrated high awareness. Logistic regression analysis identified age and educational attainment as significant predictors of awareness levels (*p* < 0.001). These results highlight a moderate understanding of irradiated foods among the Saudi population, with significant variations based on demographic factors. The study's conclusion emphasizes the necessity for tailored educational initiatives that cater to specific demographic groups to enhance understanding and awareness of irradiated food technologies in Saudi Arabia. This study thereby provides valuable insights for policymakers and health educators in designing effective communication strategies about irradiated foods.

## 1 Introduction

The global food security crisis is a pressing issue, with approximately 800 million people, or 11% of the world's population, suffering from chronic hunger, and 2 billion experiencing micronutrient deficiencies (1). If current trends continue, it is projected that around 653 million individuals will remain undernourished by 2030. Furthermore, foodborne illnesses pose a significant health risk, with an estimated 600 million people, nearly one in 10 globally, falling ill from consuming contaminated food each year, resulting in 420,000 deaths (2).

Food irradiation, a preservation process that ensures food safety, has emerged as a potential solution to these challenges. This process involves exposing food to ionizing radiation, such as gamma photons emitted by the ^60^Co radioisotope, X-rays generated by machines with a maximum energy of 5 MeV, or accelerated electrons with a maximum energy of 10 MeV (3, 4). The effects of irradiation vary depending on the type of food and the radiation dose applied (5), but it is primarily used for inhibiting budding, delaying maturation, reducing microbial load, eliminating pathogenic microorganisms, and sterilizing and disinfecting grains, cereals, fruits, and spices (6).

Public awareness plays a pivotal role in influencing various aspects of human life, including matters of food safety and technology (7). In the specific context of food irradiation, limited public awareness represents a substantial obstacle to its widespread acceptance and implementation (8, 9). Traditionally, authorities and experts have disseminated information on food safety and nutrition through controlled mass media channels (10). However, this approach often falls short in effectively engaging and educating the public. Consequently, many consumers lack information or hold misconceptions about food irradiation, frequently equating it with radioactivity (10). Previous research has demonstrated a dearth of consumer knowledge regarding food irradiation, with a significant portion of survey respondents acknowledging their unfamiliarity with irradiated foods (11, 12). This deficiency in knowledge has led to a cautious stance among consumers, highlighting the necessity for comprehensive nationwide education on food irradiation technology (10). The term “irradiation” often evokes negative perceptions due to its association with the word “radiation,” which can lead to unfounded fears and misconceptions. As a result, many consumers remain uninformed or misinformed about food irradiation, often confusing it with radioactivity (13). However, when consumers are properly informed about the real risks and benefits of food irradiation, most react positively.

Many studies have used various instruments such as surveys, focus groups, and psychometric studies to assess consumer attitudes and acceptance of new food technologies, including food irradiation. These studies aim to understand how consumers perceive the costs, benefits, and risks of food irradiation (14–17). Rusin et al. developed and validated the Awareness Scale for Consumption of Irradiated Foods (ASCIF), a psychometric instrument that measures awareness, labeling, and safety of irradiated foods in Brazil, with the potential to be adapted to other languages and cultures (18). Additionally, studies on heuristics and conjoint analysis have been used to determine consumer preferences and biases when evaluating new food technologies (19–22).

In Saudi Arabia, food security and safety are critical concerns, underscored by national policies like Saudi Vision 2030. The country's unique blend of cultural practices and religious beliefs influences food safety protocols, while its arid climate necessitates a heavy reliance on food imports, further emphasizing the need for effective food preservation technologies such as irradiation (23). Despite its approval, food irradiation is not widely understood, highlighting a notable gap in local research and knowledge. This study addresses this gap by evaluating public awareness and perceptions of irradiated foods in Saudi Arabia, thereby enriching our understanding of how such perceptions vary across different cultural and socioeconomic contexts. The findings are particularly pertinent for countries with similar characteristics to Saudi Arabia and provide a valuable comparative framework for those with differing contexts, ultimately enhancing global understanding of public perceptions toward food irradiation technology.

## 2 Materials and methods

### 2.1 Study design and population

This cross-sectional study aimed to evaluate the awareness of the Saudi Arabian population regarding the consumption of irradiated foods. The study was conducted between May 2023 and January 2024. The sampling strategy targeted a diverse geographic distribution, covering all regions across the country. The sample size for the study was calculated using G^*^Power software (24), considering an effect size of 0.15, an alpha level of 0.05, and a power of 0.95. Using G^*^Power software, it was determined that a minimum sample size of *N* = 472 was required. However, a convenience sample of 712 respondents was collected, which is considered sufficient for this study. The questionnaire was distributed online via Google Forms, and the survey link was disseminated through various networks to reach the target audience effectively.

### 2.2 Ethical considerations

The study was conducted with strict adherence to ethical standards. Informed consent was obtained electronically, ensuring participants were aware of the study's purpose, the confidentiality of their responses, and the voluntary nature of their participation. An institutional review board at Jazan University reviewed and approved the study, ensuring compliance with ethical guidelines.

### 2.3 Data collection tool

The first section of the survey collected socio-demographic variables such as age, gender, qualification, income level, work status, and marital status. The Awareness Scale for Consumption of Irradiated Foods (ASCIF) was the primary tool used, previously developed and validated (18). The ASCIF consists of a standardized, self-administered questionnaire, comprising four key factors: concepts, awareness, labeling, and safety of irradiated foods. The construct was defined as follows: Safety of Irradiated Foods focuses on concerns about the safety aspects of irradiated foods, including nutritional, chemical, physical, microbiological, and nuclear safety. Concepts covers basic definitions and principles of irradiated foods, including irradiation processes, sources, and dosages. Awareness assesses public knowledge and attitudes toward the consumption of irradiated foods, including quality perceptions and consumption preferences. Labeling examines understanding of legislation and labeling practices, including recognition of the Radura symbol and label information on irradiated foods. The ASCIF comprising 31 items: safety (15 items), Concepts (8 items), Labeling (5 items), and Awareness (3 items). Respondents rated their agreement with each statement on a five-point Likert scale, ranging from strongly disagree (1) to strongly agree (5). The mean scores for each factor were calculated, with higher scores representing a greater level of awareness. The reliability of the ASCIF, previously validated, was confirmed with a Cronbach's Alpha coefficient appropriate for this study (18).

The scoring system is segmented into three levels: “poor awareness” (scores from 1.00 to 2.33), “intermediate awareness” (scores from 2.34 to 3.66), and “high awareness” (scores from 3.67 to 5.00). These segments are based on the logical division of the scale, where the midpoint of 3 indicates a neutral stance. The range for “poor awareness” represents scores considerably below this neutral point, indicating a lower level of agreement or awareness. “Intermediate awareness” encompasses scores around the midpoint, reflecting a moderate level of awareness where respondents are neither in strong agreement nor disagreement. The “high awareness” category includes scores well above the midpoint, suggesting a higher level of agreement or awareness.

### 2.4 Data analysis

The data were analyzed using the Statistical Package for Social Sciences (SPSS), Version 27. Descriptive statistics (mean, standard deviation [SD], *n*, %) were computed for both continuous and categorical variables. The normality of data distribution was checked using the Kolmogorov–Smirnov test. Differences in ASCIF scores related to participant characteristics were examined using the Mann–Whitney U test and the Kruskal–Wallis test. Spearman correlation analysis was used to explore the relationships between the four ASCIF factors: Safety, Concepts, Labeling, and Awareness. Linear regression analysis was employed to identify predictors of overall awareness scores, considering all independent variables in one model. A *p* < 0.05 was considered statistically significant in all analyses.

## 3 Results

The study's sample consisted of 712 individuals, with a gender distribution of 46.63% males (*n* = 332) and 53.37% females (*n* = 380). The age breakdown showed 55.62% (*n* = 396) in the 18–30 years category, 26.40% (*n* = 188) in the 31–40 years bracket, and 17.98% (*n* = 128) in the 41–50 years group. In terms of education, 70.79% (*n* = 504) had a bachelor's degree or higher, 29.21% (*n* = 208) had high school education or less. Income levels were diverse, with the largest segment (42.70%, *n* = 304) earning < 5,000. Work status was divided among 37.08% students (*n* = 264), 29.78% unemployed (*n* = 212), and 33.15% employed (*n* = 236). Regarding marital status, 66.85% (*n* = 476) were single and 33.15% (*n* = 236) were married. There were no significant differences across all demographics and the overall mean score of ASCIF (Table 1).

**Table 1:** Demographic characteristics and the overall mean score of awareness scale for consumption of irradiated foods.

**Item**	**Variables**	**Count (%)**	**Overall ASCIF mean±SD**	***p*-value**
Gender	Male	332 (46.63%)	3.07 ± 0.77	0.452^a^
	Female	380 (53.37%)	2.98 ± 0.84	
Age (years)	18–30 years old	396 (55.62%)	3.00 ± 0.81	0.630^b^
	31–40 years old	188 (26.40%)	3.09 ± 0.91	
	41–50 years old	128 (17.98%)	3.28 ± 0.35	
Qualification	High school or less	208 (29.21%)	2.93 ± 0.99	0.627^a^
	Bachelor or higher	504 (70.79%)	3.06 ± 0.69	
Monthly income (SAR)	< 5,000	304 (42.70%)	3.05 ± 0.89	0.403^b^
	5,000–10,000	108 (15.17%)	3.00 ± 0.87	
	11,000–15,000	152 (21.35%)	3.20 ± 0.50	
	More than 15,000	148 (20.79%)	2.78 ± 0.81	
Work status	Student	264 (37.08%)	3.18 ± 0.70	0.253^b^
	Unemployed	212 (29.78%)	3.13 ± 0.87	
	Employed	236 (33.15%)	2.96 ± 0.88	
Marital status	Single	476 (66.85%)	2.97 ± 0.67	0.455
	Married	236 (33.15%)	3.06 ± 0.82	

^a^Mann–Whitney U test, ^b^Kruskal–Wallis test, ASCIF, Awareness Scale for Consumption of Irradiated Foods.

The ASCIF item analysis revealed diverse awareness levels and attitudes toward irradiated food among participants (Table 2). The Safety subscale showed an overall mean score of 2.87 ± 0.92, with the highest confidence in buying foods labeled as irradiated (3.08 ± 1.18) and trust in the safety of irradiated food based on its non-radioactivity and endorsements from WHO and FAO (3.01). However, willingness to consume irradiated food and perceptions of its long-term health impacts scored the lowest (2.8), indicating a threshold of acceptance contingent on safety assurances. Items of significant concern or lower acceptance included willingness to pay more for irradiated food (2.66 ± 1.1), encouraging consumption of irradiated foods (2.76 ± 1.08), and consuming irradiated foods knowing they do not cause health damage (2.7 ± 1.17).

**Table 2:** Item analysis within the consumption of irradiated foods awareness scale.

**Items**	**Mean ±SD**
**Safety**	
I have confidence in buying foods labeled as “treated by irradiation”.	3.08 ± 1.18
I would buy irradiated food because I know this process does not make the food radioactive	3.01 ± 1.11
Irradiated foods are nutritional safe	2.93 ± 1.08
I would consume irradiated food	2.93 ± 1.08
I would be willing to pay more for irradiated food	2.66 ± 1.10
I would encourage consumption of irradiated foods	2.76 ± 1.08
I would consume irradiated foods, as I know they do not cause health damage	2.70 ± 1.17
I would consume irradiated food because I know that these are safe for consumption	2.80 ± 1.11
I feel safe about the consumption of irradiated foods	2.80 ± 1.12
I approve the consumption of irradiated foods	2.80 ± 1.16
I consider that irradiated foods are not harmful to health in the short term	2.99 ± 1.11
I consider that irradiated foods are not harmful to health in the medium term	2.95 ± 1.14
I consider that irradiated foods are not harmful to health in the long term	2.81 ± 1.15
I consider that irradiated foods are not harmful to the health of future generations	2.81 ± 1.11
The World Health Organization (WHO) and the United Nations (FAO) recommend the irradiation of food	3.01 ± 1.03
Overall	2.87 ± 0.92
**Concept**	
Irradiated food is different from radioactive food	3.58 ± 1.03
Food irradiation can be used to reduce microbial load on food	3.3 ± 1.04
Irradiated food is microbiologically safe	3.01 ± 1.15
The irradiation of food can be used to inhibit the budding of bulbs, roots and tubers	3.20 ± 1.09
Food irradiation can be used to delay the ripening of fruits	2.93 ± 1.02
The minimum absorbed dose by the irradiated food must be sufficient to achieve the intended purpose	3.43 ± 1.16
Saudi Arabia authorizes the use of food irradiation	2.97 ± 1.08
Food irradiation can be used to increase shelf life	3.03 ± 1.08
Overall	3.18 ± 0.79
**Label**	
It necessary to carry out educational campaigns to inform the population about the irradiation of food	3.37 ± 1.27
All foods that undergo irradiation should have this information highlighted on the product label	3.52 ± 1.38
I consider that the additional information contained in the labels of irradiated foods is important	3.42 ± 1.30
I consider the symbol of Radura important in the labels of irradiated foods	3.41 ± 1.29
The food label should highlight the information of irradiated food	3.49 ± 1.21
Overall	3.44 ± 1.15
**Awareness**	
I consciously consume irradiated food	2.82 ± 1.15
I know some irradiated food	2.61 ± 1.15
I know Radura, the symbol used to represent irradiated food	2.62 ± 1.28
Overall	2.68 ± 1.03

The Concept subscale recorded an overall mean score of 3.18 ± 0.79, with a clear understanding of the distinction between irradiated and radioactive food (3.58 ± 1.03) and the importance of sufficient irradiation dosing (3.43 ± 1.16). However, items like the use of food irradiation to delay fruit ripening (2.93 ± 1.02) and Saudi Arabia's authorization of food irradiation (2.97 ± 1.08) showed areas of lesser understanding or awareness.

The Label subscale demonstrated strong agreement on the importance of labeling irradiated foods, with an overall mean score of 3.44 ± 1.15. The demand for clear labeling of irradiated foods was most strongly expressed, with scores ranging from 3.41 to 3.52.

The Awareness subscale showed a different trend, with an overall lower mean score of 2.68 ± 1.03, indicating less awareness or engagement with irradiated foods. Knowledge of irradiated foods and recognition of the Radura symbol were the lowest with mean scores of 2.61 ± 1.15 and 2.62 ± 1.28, respectively.

In Table 3 spearman correlation matrix for the ASCIF constructs, significant correlations are observed among safety, concept, labeling, and awareness mean scores. A strong correlation between Safety and Concept (r = 0.7, *p* < 0.001) suggests that increased safety perceptions are closely linked to a better understanding of concepts behind irradiated foods. Safety also shows a moderate correlation with Labeling (r = 0.39, *p* < 0.001) and a strong correlation with Awareness (r = 0.71, *p* < 0.001), indicating that higher safety perceptions are associated with improved labeling knowledge and overall awareness. Concept and Labeling are significantly correlated (r = 0.61, *p* < 0.001), as are Concept and Awareness (r = 0.48, *p* < 0.001), demonstrating that a deeper understanding of concepts enhances labeling knowledge and awareness. The correlation between Labeling and Awareness is positive but weaker (r = 0.21, *p* < 0.01), suggesting a less pronounced but significant link between these aspects. These results highlight the interconnectedness of safety, concept, labeling, and awareness in shaping consumer perceptions about irradiated foods.

**Table 3:** Spearman correlation matrix of the awareness scale for consumption of irradiated foods (ASCIF) subscale mean scores.

**Correlation matrix**	**Safety**		**Concept**		**Labeling**		**Awareness**
Safety	—						
Concept	0.7	^***^	—				
Labeling	0.39	^***^	0.61	^***^	—		
Awareness	0.71	^***^	0.48	^***^	0.21	^**^	—

^*^p < 0.05, ^**^p < 0.01, ^***^p < 0.001.

**Figure 1:**
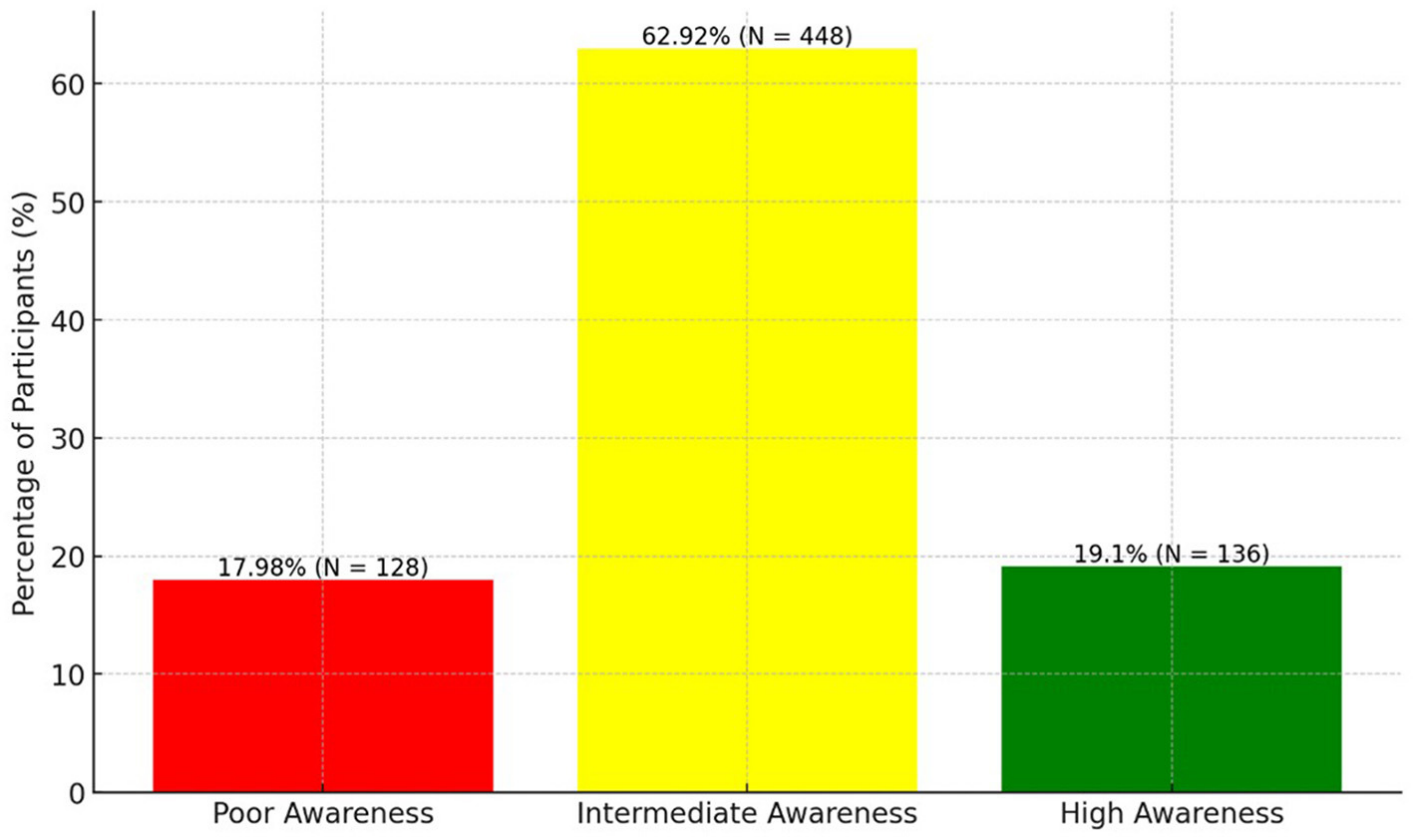
Distribution of awareness levels on irradiated foods among study participants.

The overall mean score for the ASCIF was 3.02 ± 0.81, placing the majority of participants (62.92%, 448 individuals) in the “intermediate awareness” category, indicative of a moderate understanding of irradiated foods. However, a notable portion of the sample (17.98%, 128 individuals) fell into the “poor awareness” bracket, signaling a significant gap in knowledge and perception. Conversely, 19.10% (136 individuals) achieved scores above 3.66, categorizing them in the “High Awareness” range, denoting a well-developed understanding. This distribution underscores the necessity for targeted educational efforts to enhance the overall awareness and comprehension of irradiated foods among the population (Figure 1).

The logistic regression analysis from Table 4 elucidates the influence of demographic factors on awareness levels about irradiated foods, revealing that age is a pivotal predictor. Specifically, individuals in the 41–50 age group are significantly more likely to exhibit higher awareness levels, both in the high (*p* < 0.001) and intermediate categories (*p* < 0.001), compared to the 18–30 age group. Additionally, higher educational attainment, specifically holding a bachelor's degree or higher, is associated with an increased likelihood of being in the intermediate awareness category (p = 0.013). In contrast, other demographics such as gender, income level, work status, and marital status do not show a significant impact on awareness levels. This analysis underscores the crucial role of age and education in shaping awareness about irradiated foods, highlighting the need for age-specific and educationally targeted awareness initiatives.

**Table 4:** Linear regression analysis of the awareness scale for consumption of irradiated foods (ASCIF) levels and demographic variables.

**Awareness levels**	**Predictor**	**Estimate**	**SE**	**Z**	***p***
High level—poor Level	Intercept	−1.35	0.92	−1.47	0.143
	**Gender**				
	Female—male	−0.06	0.57	−0.1	0.922
	**Age**				
	31–40 years old−18–30 years old	1.68	0.97	1.73	0.083
	41–50 years old−18–30 years old	1.45	0.67	2.03	< 0.001
	**Qualification**				
	Bachelor and above—high school or less	0.73	0.57	1.27	0.204
	**Income level**				
	11,000–15,000— < 5,000	−0.59	1.31	−0.45	0.652
	5,000–10,000— < 5,000	1.34	1.04	1.28	0.199
	More than 15,000— < 5,000	1.09	1	1.09	0.277
	**Work status**				
	Student—employed	1.25	0.83	1.51	0.132
	Unemployed—employed	0.31	0.87	0.36	0.718
	**Marital status**				
	Married—single	−0.3	0.78	−0.39	0.698
Intermediate—poor level	Intercept	−0.32	0.72	−0.45	0.655
	**Gender**				
	Female—male	−0.08	0.47	−0.18	0.86
	**Age**				
	31–40 years old−18–30 years old	0.09	0.8	0.12	0.908
	41–50 years old−18–30 years old	1.97	0.67	2.32	< 0.001
	**Qualification**				
	Bachelor and above—high school or less	1.18	0.48	2.47	0.013
	**Income level**				
	11,000–15,000— < 5,000	−0.5	1.01	−0.49	0.621
	5,000–10,000— < 5,000	1.45	0.89	1.63	0.104
	More than 15,000— < 5000	0.05	0.92	0.05	0.96
	**Work status**				
	Student—employed	1.03	0.66	1.55	0.122
	Unemployed—employed	0.1	0.67	0.15	0.881
	**Marital status**				
	Married—single	0.73	0.59	1.25	0.212

## 4 Discussion

This study aimed to evaluate public knowledge, attitudes, and awareness regarding irradiated foods in Saudi Arabia, employing the ASCIF scale across four subscales: safety, concept, label, and awareness. The significance of this research lies in its potential to inform public health strategies and regulatory policies by assessing the current level of understanding and acceptance of food irradiation among the Saudi population. With an overall mean ASCIF score of 3.02 ± 0.81, the majority of participants were categorized at an “intermediate level” of awareness, indicating a moderate but varied understanding of irradiated foods.

In this study, the results revealed insights across the four ASCIF subscales—safety, concept, label, and awareness. The safety subscale indicated a moderate confidence in irradiated food's non-radioactivity and safety endorsements by organizations such as WHO and FAO, but showed reluctance toward consuming irradiated food and concerns about long-term health effects, with a notable hesitance toward paying more for such foods. The concept subscale demonstrated a clear understanding of irradiation's technical aspects and its distinction from radioactivity, yet it also highlighted gaps in knowledge about its applications and regulatory status. Strong agreement in the label subscale emphasized the importance of clear labeling for irradiated foods, reflecting a significant demand for transparency. However, the awareness subscale showed lower overall engagement and recognition of irradiated foods and the Radura symbol, indicating a need for enhanced public education and awareness efforts. These findings suggest a moderate level of public understanding and acceptance of food irradiation, paired with identified areas for improvement in knowledge dissemination and awareness-raising to better align public perceptions with scientific and regulatory standards.

Previous studies identified several key factors contribute to the lack of knowledge or misconceptions about food irradiation. There is generally low awareness and familiarity among consumers, many of whom have never heard of the process, leading to significant knowledge gaps (11). Misconceptions about the safety and nutritional quality of irradiated foods are prevalent, often fueled by incorrect beliefs that such foods are radioactive or nutritionally depleted (11). Additionally, insufficient or inappropriate labeling hinders informed consumer choices, as many are unaware of consuming irradiated foods due to lack of clear labeling (25). The term “irradiation” itself often triggers negative perceptions linked to fears of radiation and nuclear accidents, impacting acceptance negatively unless countered by effective education. Moreover, the credibility and trustworthiness of information sources play a crucial role, with skepticism toward regulators and the food industry affecting acceptance of food technologies. Cultural and social norms vary, with some cultures showing more resistance to new food technologies, highlighting how social dynamics can shape individual perceptions.

The Spearman correlation matrix reveals significant relationships among the ASCIF constructs of safety, concept, labeling, and awareness. There is a strong correlation between safety perceptions and understanding of irradiated food concepts (r = 0.7, *p* < 0.001), with safety also moderately and strongly correlated with labeling (r = 0.39, *p* < 0.001) and awareness (r = 0.71, *p* < 0.001), respectively. This suggests that higher safety perceptions enhance both labeling knowledge and awareness. Furthermore, concept understanding is significantly linked to both labeling knowledge (r = 0.61, *p* < 0.001) and awareness (r = 0.48, *p* < 0.001), while the relationship between labeling and awareness is positive though weaker (r = 0.21, *p* < 0.01), indicating that these constructs are interrelated and collectively influence consumer attitudes toward irradiated foods. Previous research has shown significant correlations among various factors: safety and concept had a high correlation, as did safety and awareness, and concept and awareness. However, these factors all had low correlations with labeling. This suggests that while safety, concepts, and awareness regarding irradiated foods are interlinked, labeling does not show a strong correlation with these aspects (18). Such correlations also align with findings from a previous study (26), which suggest that education can significantly shift consumer attitudes toward food irradiation, especially given the prevailing lack of knowledge about its benefits. This emphasizes the need for dissemination of information regarding irradiation technologies (18), as the minimal correlation observed with labeling likely stems from a general unfamiliarity with the irradiation process and the Radura symbol (18). Demographic factors displayed varied influences on ASCIF levels. The logistic regression analysis identified age, particularly the 41–50 years group, and higher education as significant predictors of higher awareness levels. This emphasizes the importance of focusing educational initiatives on specific age groups and leveraging educational platforms to enhance understanding of irradiated foods.

The findings from this study have a long-term implication for public health policies and consumer education in Saudi Arabia. Enhanced public education could lead to increased acceptance and use of food irradiation, which, in turn, could contribute to reducing foodborne illnesses and improving food security in the region. Establishing regulatory frameworks that support clear labeling and public education on irradiated foods can facilitate more informed consumer choices.

Effective education about food irradiation involves a multifaceted approach. Key strategies include focusing communications on the safety and benefits of irradiation while directly addressing common misconceptions (8, 27, 28). Utilizing credible sources like health authorities and academics is crucial, as these are more trusted than industry or government sources (8, 29, 30). Increasing consumer familiarity through awareness campaigns and clear labeling is essential to overcome barriers of unfamiliarity. Tailoring messaging to different consumer demographics and psychographics ensures relevance and effectiveness. Emphasizing informed choice through mandatory labeling regulations also plays a vital role in increasing acceptance. Incorporating two-way engagement, especially through social media platforms, allows for integrating consumer feedback, enhancing the credibility and relevance of the communication. Overall, transparent, respectful, and targeted communication strategies, combined with appropriate regulatory measures, are key to gradually increasing familiarity and acceptance of food irradiation (31).

### 4.1 Strengths, limitations, and future directions

This study's strength lies in its use of the validated Psychometric scale, which not only allows for robust measurement of various aspects of public perception but also enables comparisons with similar studies conducted in other regions using the same scale. Additionally, this research represents the first of its kind in Saudi Arabia, providing an overview of local perceptions toward food irradiation. However, the study's cross-sectional design and convenience sampling method introduce limitations that may affect the generalizability of the results. While a sample of 712 respondents provides initial insights, the small size and method of recruitment—through various social media networks—might not fully represent the broader Saudi population. There is potential for selection bias, as participants with a pre-existing interest in food safety topics, such as irradiation, may have been more likely to respond. Future research could address these limitations by employing a longitudinal design to track changes in perception over time and using random sampling to enhance representativeness.

## 5 Conclusion

The study revealed a diverse spectrum of awareness, with the majority of participants (62.92%) exhibiting intermediate awareness, while 17.98% displayed poor awareness, and 19.10% demonstrated high awareness. The overall mean score for the ASCIF was 3.02 ± 0.81. Individuals aged 41–50 and those with higher education were more likely to have higher awareness. This study emphasizes the necessity for tailored educational initiatives that cater to specific demographic groups to enhance understanding and awareness of irradiated food in Saudi Arabia. This study thereby provides valuable insights for policymakers and health educators in designing effective communication strategies about irradiated foods.

## Data availability statement

The raw data supporting the conclusions of this article will be made available by the authors, without undue reservation.

## Ethics statement

The studies involving humans were approved by the Ethics Committee at Jazan University, Saudi Arabia. The studies were conducted in accordance with the local legislation and institutional requirements. The participants provided their written informed consent to participate in this study.

## Author contributions

NS: Conceptualization, Data curation, Formal analysis, Funding acquisition, Investigation, Methodology, Project administration, Resources, Software, Supervision, Validation, Visualization, Writing – original draft, Writing – review & editing.
